# Characterization of the flanking region of the Shiga toxin operon in Stx2a bacteriophages reveals a diversity of the NanS-p sialate O-acetylesterase gene

**DOI:** 10.3934/microbiol.2023030

**Published:** 2023-08-02

**Authors:** Stefanía B. Pascal, Ramiro Lorenzo, María Victoria Nieto Farías, John W.A. Rossen, Paula M. A. Lucchesi, Alejandra Krüger

**Affiliations:** 1 Universidad Nacional del Centro de la Provincia de Buenos Aires (UNCPBA), Facultad de Ciencias Veterinarias, CISAPA, Tandil, Buenos Aires, Argentina; 2 Centro de Investigación Veterinaria de Tandil (CIVETAN), UNCPBA-CICPBA-CONICET, Tandil, Buenos Aires, Argentina; 3 Laboratory of Neurophysiology, ULB Neuroscience Institute, Université Libre de Bruxelles (ULB), Brussels, Belgium; 4 Laboratory of Medical Microbiology and Infectious Diseases, Isala Hospital, Zwolle, The Netherlands; 5 Department of Medical Microbiology and Infection Prevention, University of Groningen, University Medical Center Groningen, Groningen, The Netherlands

**Keywords:** Shiga toxin, Stx subtypes, phage, sialate O-acetylesterase, late genomic region, diversity

## Abstract

Shiga toxin-producing *E. coli* (STEC) are diarrheagenic strains that can cause bloody diarrhea and hemolytic-uremic syndrome. Their main virulence factor, the Shiga toxin (Stx), is encoded by phages integrated into the bacterial chromosome. Stx phages are widely diverse and carry many genes with limited or unknown function. As the toxin subtype Stx2a is associated with highly pathogenic strains, this study was mainly focused on the characterization of the *stx* flanking region of Stx2a phages. Of particular interest was a sialate O-acetylesterase (NanS-p), which has been described previously to be encoded downstream *stx* in some phage genomes and may confer a growth advantage for STEC. Complete DNA sequences of Stx2a phages and prophages were retrieved from the GenBank database, and the genomic regions from anti-terminator Q to holin S genes were bioinformatically analyzed. Predicted NanSp sequences from phages encoding other Stx subtypes were also studied. Additionally, expression of *nan*S-p was quantified by qPCR in strains selected from our laboratory collection. The analysis of Stx2a phage genomes showed that all carried the *Q*, *stx*_2a_, *nan*S-p and *S* genes, but with allele diversity and other sequence differences. In particular, sequence differences were detected in each of the three domains of NanS-p esterases encoded by Stx2a phages and other Stx phages; however, *nan*S-p was not identified in the Stx2e, Stx2f and Stx2g phages analyzed. The expression of *nan*S-p increased in most *stx*_2a_-positive strains under phage inducing conditions, as was previously shown for *stx*_2a_. As the present work showed diversity at the Q-S region among Stx phages, and particularly in the encoded NanS-p enzyme, future studies will be necessary to evaluate if NanS-p variants differ in their activity and to assess the impact of the absence of *nan*S-p in certain Stx phages.

## 1. Introduction

*Escherichia coli* is an abundant member of the gastrointestinal microbiota of mammals. Most *E. coli* strains are harmless organisms that efficiently colonize the mucous layer of the mammalian colon [1]. However, some *E. coli* clones have acquired the ability to cause diseases and adapt to new niches. The genetic information providing metabolic and pathogenic properties for adaptation to particular environmental conditions is frequently encoded on mobile genetic elements, such as bacteriophages, plasmids and transposons [1],[2].

According to the virulence factors acquired, pathogenic *E. coli* strains involved in diarrheal diseases have been classified into several pathotypes. The Shiga toxin-producing *E. coli* (STEC) pathotype represents the group of *E. coli* strains whose virulence hallmark is the production of Shiga toxins (Stx). These highly pathogenic strains can cause severe human diseases, such as bloody diarrhea (BD) and hemolytic-uremic syndrome (HUS) [3]. Stx toxins are among the most potent bacterial toxins known, with rRNA-N-glycosidase activity that inactivates 60S ribosomal subunits and disrupts protein synthesis in eukaryotic cells [4],[5]. Shiga toxins are classified into two major types: Stx1 and Stx2, with several subtypes. STEC strains harboring the *stx*_2a_ subtype have shown the highest rates of HUS, hospitalization and BD [3].

The genes encoding the Stx toxins are carried by bacteriophages (Stx phages) integrated into the bacterial chromosome. These phages are usually called lambda-like because the analyses of the first genomes sequenced showed a similar genetic organization (comprising recombination, early regulation, replication, late regulation, lysis and structural gene regions) and many homologous genes to those of the bacteriophage lambda [6]–[8]. However, the Stx phage family is a diverse group of phages, variable in the *stx* subtype they carry and in genome size, genetic composition and virion morphology [7],[9],[10]. Furthermore, Stx phages carry many genes whose function is not yet well-known [8],[11]–[13].

The *stx* genes are located in the late transcribed region, downstream of the Q protein-encoding gene and upstream of the lysis cassette. Thus, *stx* expression is controlled by the anti-terminator Q protein, which allows transcription to continue into late genes [10],[14]. Under inducing conditions, transcription of *stx* and lysis genes is Q-activated, resulting in host cell lysis and toxin release [15].

Stx2a-encoding phages (Stx2a phages) are the most studied prophages of STEC, as they are located in the genomes of the strains most frequently associated with severe clinical outcomes. Studies performed on groups of Stx2a phages show a high level of genomic diversity also within the Stx2a phage family. For example, differences among Stx2a phages from O157:H7 STEC strains were observed in genes in the replication and early regulatory regions [9],[16], and researchers have identified distinct types among this group of phages [16]–[18]. Noteworthy, Yin et al. [16] and Krüger et al.[12] found that PST2, one of these phage types detected within O157:H7 strains associated with a high incidence of HUS, was closely related to Stx2a phages identified in some non-O157 STEC strains corresponding to serotypes like O103:H2, O104:H4 and O145:H-.

Phages, including Stx phages, have played a significant role in the evolution and diversity of STEC strains [17]–[19]. However, little is known about the impact of these phages on the fitness and virulence of the lysogens. As mentioned above, Stx phages are essential in regulating Shiga toxin production. In addition, various studies have demonstrated that lysogenic infection by specific Stx phages produced a significant impact on host gene expression. It was found that carriage of phage ΦMin27 (Δstx::cat) in *E. coli* K-12 MG1655 had a direct effect on the global expression of bacterial genes, and an increase of acid tolerance [20], integration of φ24B (Δstx::kan) into *E. coli* K-12 MC1061 increased the rates of respiration, cell proliferation and acid resistance [21],[22]. In addition, lysogenic infection by Stx2a phages φO104 and φPA8 introduced dramatic changes in carbon source utilization of *E. coli* MG1655 [23], and lysogenization of *E. coli* K-12 by Sp5 decreased cell motility under anaerobic conditions [24]. Moreover, it has been shown that NanS-p esterases encoded by Stx phages, but also by phages not containing *stx* genes, conferred a growth advantage to O157 EDL933 and O104 LB226692 on 5-N-acetyl-9-O-acetyl neuraminic acid as a carbon source [25],[26].

Considering that the Stx2a phage family is a group of diverse members, this study aimed to perform a comprehensive analysis of the *stx* flanking region of Stx2a phages belonging to several serotypes and origins, giving particular emphasis on the characterization of the predicted NanS-p esterase sequences.

## 2. Material and methods

### 2.1. Analysis of stx flanking region of Stx2a phage genomes

DNA sequences of Stx2a phages and prophages were retrieved from the GenBank database using the BLASTN program (https://www.ncbi.nlm.nih.gov/blast/) [27] with the *stx*_2a_ sequence from the phage 933W (X07865) as a query sequence. Sequences were downloaded either directly as complete phage genomes or retrieved from bacterial host genomes by analysis with PHAge Search Tool Enhanced Release (PHASTER) web server (http://phaster.ca) [28] and subsequently analyzed with the VirulenceFinder web server (https://cge.food.dtu.dk/services/VirulenceFinder/) [29]. This phage database of complete Stx2a phage genomes was created in August 2019.

The nucleotide sequences from anti-terminator Q to holin S encoding genes (named *Q*-*S* region) were extracted and aligned with the BLASTN program using the corresponding sequence of the phage 933W (AF125520: 20205-26045) as the query sequence. Insertion sequence elements were identified by comparing the sequences against the ISfinder database (https://isfinder.biotoul.fr/blast.php) [30]. The ORFs were determined by examination of annotations at GenBank and using ORFinder (https://www.ncbi.nlm.nih.gov/orffinder/). Protein domains were annotated using the Conserved Domain Search Service (CD-Search) (https://www.ncbi.nlm.nih.gov/Structure/cdd/wrpsb.cgi) [31] and InterProScan (https://www.ebi.ac.uk/interpro/search/sequence/) [32]. A search for close homologs of the holin sequences was performed with TC-BLAST at the Transporter Classification Database (TCD) (https://www.tcdb.org/progs/blast.php) [33]. Distance trees were produced using BLAST pairwise alignment (https://www.ncbi.nlm.nih.gov/blast/) [27]. Alignments for *Q*-*S* region were generated with progressive MAUVE [34] using default settings. Whole phages were aligned with the exception of the integrated Stx2a phages where *Q*-*S* region was selected.

Sequence alignments of NanS-p and corresponding domains were performed using Muscle implemented in MEGA [35] . Sequences sharing 100% identity were represented by only one of them to simplify comparisons. C-terminal variants were classified based on an identity threshold of 80% after alignment with Clustal Omega using default parameters [36].

### 2.2. Relative quantification of nanS-p expression in stx_2a_-positive STEC strains

#### 2.2.1. Sample collection, RNA extraction and cDNA synthesis

A group of 13 *stx*_2a_-positive STEC strains, whose genomes have been sequenced (Illumina MiSeq sequencing platform as described by [12]), were selected from our collection (Supplementary Table 1). The reference strain *E. coli* O157:H7 EDL933 was also used. Expression of *nan*S-p was quantified in mitomycin-C-induced STEC cultures relative to uninduced cultures using real-time qPCR. Bacterial growth conditions, total RNA extraction and cDNA synthesis were previously described for *stx*_2a_-expression of the same strains [37].

#### 2.2.2. Primers and qPCR conditions

Primers DS-forward (5′-CCTTATGGTAGTGCGCTGATT-3′) and DS-reverse (5′-AGTCCCTCACCGTATGACA-3′) were designed to allow the amplification of the DUF-SASA region of *nan*S-p subtypes present mainly in Stx prophages but not in bacterial *nan*S. In order to check *in silico* if the region amplified by these primers could be present in other genomic regions beyond those present in Stx2a prophages, a search was done with the BLASTN program using the sequence of the PCR product for the DUF-SASA region of phage 933W against the genome sequences of the 13 STEC strains listed in Supplementary Table 1.

Each reaction contained 4 µL of 1/10 diluted cDNA sample, 10 µL of 2X SYBR Green Master Mix (FastStart Universal SYBR Green Master, Roche) and either the pair of primers DS-forward/DS-reverse (400 nM each) to amplify *nan*S-p, or primers TufAqR/TufAqF (300 nM each) to amplify the housekeeping gene *tufA* [38]. The amplification and detection of the specific products were carried out in an OneStep Plus Real-Time PCR System cycler, with the following amplification conditions: 2 min at 50 °C, 10 min at 95 °C and 40 cycles of 20 s at 95 °C and 60 s at 60 °C. Standard curves were made with serial dilutions of a pool of cDNA samples. The real-time RT-PCR efficiency for each gene was determined by a linear regression model according to the equation: E = 10^[−1/slope]^. The *nan*S-p expression levels for mitomycin C-induced cultures were calculated relative to non-induced ones by the ΔΔCT method [39] using the efficiency corresponding to each gene.

**Table 1.** Information on Stx2a phage and prophage sequences used for Q-S analysis

Accession number^†^	Phage name	STEC host					Best matching *stx* sequence*	Stx2a variant^#^
		Name	Serotype	Source	Isolation place	Year or period		
AF125520	933W	EDL933	O157:H7	NA	NA	NA	X07865	Stx2a-O157-EDL933
CP001368	NA	TW14359	O157:H7	human	USA	2006	X07865	Stx2a-O157-EDL933
CP001164	NA	EC4115	O157:H7	human	USA	2006	X07865	Stx2a-O157-EDL933
AP012529.1	Stx2a_F403	F403	O157:H7	human	Japan	1990s	X07865	Stx2a-O157-EDL933
AP012531.1	Stx2a_F422	F422	O157:H7	human	Japan	1990s	X07865	Stx2a-O157-EDL933
AP012532.1	Stx2a_F451	F451	O157:H7	human	Japan	1990s	X07865	Stx2a-O157-EDL933
EU311208.1	ΦMin27	Min27	O157:H7	piglet	China	NA	X07865	Stx2a-O157-EDL933
AP004402.1	Stx2φ-I	Okayama O-27	O157:H7	human	Japan	1996	X07865	Stx2a-O157-EDL933
KP682381.1	PA28	PA28	O157:H7	human	USA	2006-2008	X07865	Stx2a-O157-EDL933
AP012533.1	Stx2a_F723	F723	O157:H7	human	Japan	1990s	X07865	Stx2a-O157-EDL933
AP012535.1	Stx2a_WGPS9	WGPS9	O157:H7	human	Japan	1998	X07865	Stx2a-O157-EDL933
CP027390	NA	2015c-4944	O26:H11	NA	USA	NA	X07865	Stx2a-O157-EDL933
AP010958	NA	12009	O103:H2	human	Japan	2001	X07865	Stx2a-O157-EDL933
KP682376.1	PA12	PA12	O157:H7	human	USA	2006-2008	X07865	Stx2a-O157-EDL933
KP682391.1	PA51	PA51	O157:H7	human	USA	2006-2008	X07865	Stx2a-O157-EDL933
CP027599	NA	97-3250	O26:H11	human	NA	1997	X07865	Stx2a-O157-EDL933
JQ011318.1	TL-2011c	NVH-734	O103:H25	human	Norway	2006	AB030484	Stx2a-O157-EDL933
CP027347	NA	2013C-4361	O111:H8	human	USA	2013	X07865	Stx2a-O157-EDL933
AP000363.1	VT2-Sakai	Sakai	O157:H7	NA	Japan	1996	X07865	Stx2a-O157-EDL933
CP006027	NA	RM13514	O145:H28	human	USA	2010	X07865	Stx2a-O157-EDL933
CP027362	NA	95-3192	O145:H28	NA	USA	NA	AB030484	Stx2a-O157-EDL933
CP027221	NA	2015c-3101	O111:H8	human	USA	2014	X07865	Stx2a-O157-EDL933
CP027317	NA	2015c-3107	O121:H19	human	USA	2014	AB030484	Stx2a-O157-EDL933
HE664024.1	P13374	CB13374	O104:H4	sprouts seeds	Germany	2011	AB030484	Stx2a-O157-EDL933
HG803182.1	P13363	CB13363	O104:H4	*Cucumis sativus*	Germany	2011	AB030484	Stx2a-O157-EDL933
HG792102.1	P13803	CB13803	O2:H27	bovine feces	Germany	2011	AB030484	Stx2a-O157-EDL933
HG792105.1	P14437	CB14437	O104:H4	human	Norway	2006	AB030484	Stx2a-O157-EDL933
HG792103.1	P8983	CB8983	O104:H4	human	Germany	2001	AB030484	Stx2a-O157-EDL933
KU298437.1	phiON-2011	ON-2011	O104:H4	NA	NA	NA	AB030484	Stx2a-O157-EDL933
HG792104.1	P13771	2009EL-2050	O104:H4	human	Georgia	2009	AB030484	Stx2a-O157-EDL933
AP012534.1	Stx2a_F765	F765	O157:H7	human	Japan	1990s	X07865	Stx2a-O157-EDL933
KY914478.1	ArgO145	FB5	O145:H-	bovine feces	Argentina	2001-2002	X07865	Stx2a-O157-EDL933
HQ424691.1	VT2_phi272	71074	O157:H7	NA	NA	NA	X07865	Stx2a-O157-EDL933
KP682371.1	PA2	PA2	O157:H7	human	USA	2006-2008	X07865	Stx2a-O157-EDL933
KP682374.1	PA8	PA8	O157:H7	human	USA	2006-2008	X07865	Stx2a-O157-EDL933
KF971864.1	phi191	ED 191	O111:H2	human	France	1992	AB030484	Stx2a-O157-EDL933
AP005154.1	II DNA	Morioka V526	O157:H7	human	Japan	1996	X07865	Stx2a-O157-EDL933
KP682372.1	PA4	PA4	O157:H7	human	USA	2006-2008	X07865	Stx2a-O157-EDL933
KP682373.1	PA5	PA5	O157:H7	human	USA	2006-2008	X07865	Stx2a-O157-EDL933
KP682375.1	PA11	PA11	O157:H7	human	USA	2006-2008	X07865	Stx2a-O157-EDL933
KP682377.1	PA16	PA16	O157:H7	human	USA	2006-2008	X07865	Stx2a-O157-EDL933
KP682378.1	PA18	PA18	O157:H7	human	USA	2006-2008	X07865	Stx2a-O157-EDL933
KP682379.1	PA21	PA21	O157:H7	human	USA	2006-2008	X07865	Stx2a-O157-EDL933
KP682380.1	PA27	PA27	O157:H7	human	USA	2006-2008	X07865	Stx2a-O157-EDL933
KP682382.1	PA29	PA29	O157:H7	human	USA	2006-2008	X07865	Stx2a-O157-EDL933
KP682383.1	PA30	PA30	O157:H7	human	USA	2006-2008	X07865	Stx2a-O157-EDL933
KP682384.1	PA32	PA32	O157:H7	human	USA	2006-2008	X07865	Stx2a-O157-EDL933
KP682385.1	PA33	PA33	O157:H7	human	USA	2006-2008	X07865	Stx2a-O157-EDL933
KP682386.1	PA36	PA36	O157:H7	human	USA	2006-2008	X07865	Stx2a-O157-EDL933
KP682387.1	PA42	PA42	O157:H7	human	USA	2006-2008	X07865	Stx2a-O157-EDL933
KP682388.1	PA44	PA44	O157:H7	human	USA	2006-2008	X07865	Stx2a-O157-EDL933
KP682389.1	PA45	PA45	O157:H7	human	USA	2006-2008	X07865	Stx2a-O157-EDL933
KP682390.1	PA50	PA50	O157:H7	human	USA	2006-2008	X07865	Stx2a-O157-EDL933
KP682392.1	PA52	PA52	O157:H7	human	USA	2006-2008	X07865	Stx2a-O157-EDL933
AP010960	NA	11128	O111:H-	human	Japan	2001	AF524944	Stx2a-O157-SF-258-98
CP018237	NA	155	O157:H7	human	United Kingdom	2012	AF524944	Stx2a-O157-SF-258-98
CP006262	NA	RM13516	O145:H28	human	Belgium	2007	AF524944	Stx2a-O157-SF-258-98
CP027584^‡^	NA	00-3076	O113:H21	human	USA	2000	GQ429170	Stx2a-O113-TS17-08
NC_008464	86	DIJ1	O86:H-	NA	Japan	NA	X07865	Stx2a-O157-EDL933

NA: not available in database record or associated reference. † Accession number to phage genome or bacterial genome where the prophage sequence was obtained. *Best matching *stx* sequence according to VirulenceFinder analyses. ^#^ Stx2a variant was assigned in correspondence to the aa variant encoded by the best matching nucleotide sequence, according to the study of Scheutz et al. (2012) [40]. ^‡^ This strain contains two *stx*_2a_ copies, in positions 926400-927640 and 2110659-2111899. The phage that carried the first copy was used for the study.

**Table 2.** Characteristics of different NanS-p subtypes encoded by Stx2a phages

Amino acid sequence number	Protein length (aa)	Number of phages encoding the sequence	DUF1737 variant	SASA variant	Variable amino acids in SASA*	C-terminal region	Accession numbers of the corresponding phage or bacterial genomic sequence
1	645	11	D 1	SASA 1	DFSVEFGVA	CTR 1	AF125520, CP001368, CP001164, AP012529, AP012531, AP012532, EU311208, AP004402, KP682381, AP012533, AP012535
2	645	2	D 1	SASA 2	DFSV**D**FGVA	CTR 1	CP027390, AP010958
3	643	2	D 1	SASA 1	DFSVEFGVA	CTR 1	KP682376, KP682391
4	645	1	D 1	SASA 2	DFSV**D**FGVA	CTR 1	CP027599
5	645	1	D 2	SASA 3	DFS**A**EFGVA	CTR 1	JQ011318
6	645	1	D 1	SASA 1	DFSVEFGVA	CTR 1	CP027347
7	645	1	D 1	SASA 4	DFSVEFG**A**A	CTR 1	AP000363
8	645	1	D 1	SASA 2	DFSV**D**FGVA	CTR 1	CP006027
9	645	1	D 2	SASA 3	DFS**A**EFGVA	CTR 1	CP027362
10	645	1	D 2	SASA 5	DFS**A**E**LDA**A	CTR 1	CP027221
11	645	1	D 2	SASA 6	DFS**A**EFG**A**A	CTR 1	CP027317
12	645	6	D 2	SASA 3	DFS**A**EFGVA	CTR 1	HE664024, HG803182, HG792102, HG792105, HG792103, KU298437
13	645	1	D 2	SASA 3	DFS**A**EFGVA	CTR 1	HG792104
14	645	5	D 2	SASA 7	DFS**A**EF**DGT**	CTR 1	AP012534, KY914478, HQ424691, KP682371, KP682374
15	461	1	D 2	SASA 3	DFS**A**EFGVA	CTR 2	KF971864
16	634	18	D 1	SASA 1	DFSVEFGVA	CTR 3	AP005154, KP682372, KP682373, KP682375, KP682377, KP682378, KP682379, KP682380, KP682382, KP682383, KP682384, KP682385, KP682386, KP682387, KP682388, KP682389, KP682390, KP682392
17	645	1	D 3	SASA 8	**EYRA**EFG**A**A	CTR 1	AP010960
18	645	2	D 3	SASA 9	**EYRA**EF**DGT**	CTR 1	CP018237, CP006262
19	645	1	D 3	SASA 10	**EYRA**EFGVA	CTR 1b	CP027584
20	631	1	D 2	SASA 11	DFS**AQLKA**A**	CTR 4	NC_008464

* Positions correspond to amino acids 100, 102, 104, 124, 209, 241 253, 257 and 275 in 933W NanS-p sequence. Differences with SASA1 are highlighted in bold. ** Differences were not only restricted to these positions.

#### 2.2.3. Characterization of NanS-p encoded by other Stx phages

In order to investigate if NanS-p is encoded by phages carrying *stx* subtypes other than *stx*_2a_, *stx*-related sequences were identified in the GenBank DNA database using the BLASTN program with reference sequences for *stx*_1a_, *stx*_1c_, *stx*_2b_, *stx*_2c_, *stx*_2d_, *stx*_2e_, *stx*_2f_ and *stx*_2g_ [40] as query sequences. Similar analyses to those conducted for Stx2a phages were performed to construct a database of phages encoding these subtypes and evaluate the characteristics of *nan*S-p.

## 3. Results

### 3.1. Analysis of stx flanking region in Stx2a phage genomes

Fifty-nine DNA sequences of Stx2a (pro)phages were obtained for the study (Table 1). The available information on bacterial hosts indicates that they belonged to ten serogroups and were generally isolated from human samples in several countries.

**Figure 1.**
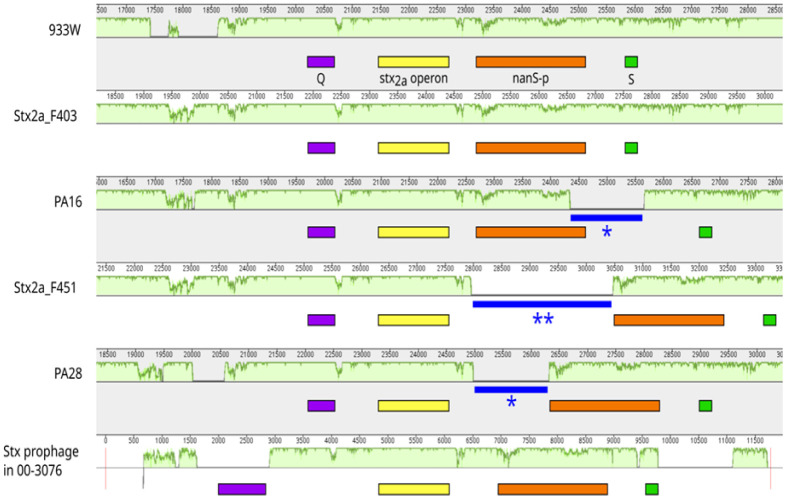
Comparison of the *Q*-*S* region of Stx2a phages representative of the major differences detected. Similarity profiles were generated with MAUVE (method details in [34]). Each horizontal panel represents each input sequence. The height of the similarity profile (in light green) corresponds to the average level of sequence conservation in that region (from 0 to 100%). The genomic position for each phage is displayed above each similarity profile except for Stx prophage in 00-3076 where positions are relative to the extracted sequence which is delimited with red lines (Q-S region in *Escherichia coli* strain 00-3076 spans from 921563 to 933340). IS elements are indicated with * (IS3 family) or ** (IS66 family).

The analysis of *stx* flanking regions confirmed that all Stx2a (pro)phages shared similar gene organization: *Q*, *stx*, *nan*S-p and *S* genes and showed 94 to 100% sequence identity with the corresponding region of 933W. Main differences were observed upstream of the operon *stx* and in the region between *stx* to *nan*S-p (including the last gene in some cases) (Figure 1). Presence of insertion sequence (IS) elements contributed to the differences in some sequences. The IS elements were located within the *nan*S-p gene or between *stx* and *nan*S-p genes (Figure 1). They corresponded to the IS3 family (significant alignments with IS1203 and IS629) except the IS in phage F451, which best matched with ISEc8 of the IS66 family.

**Figure 2.**
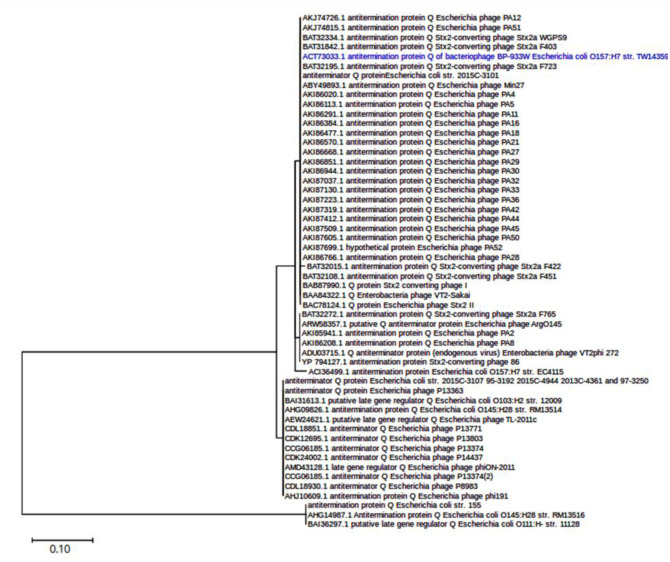
Phylogenetic tree of Q proteins encoded by Stx2a phages. The evolutionary history was inferred by using the Maximum Likelihood method and JTT matrix-based model [62], conducted in MEGA X [63]. The tree is drawn to scale, with branch lengths measured in the number of substitutions per site (scale is shown). This analysis involved 54 amino acid sequences. The sequence WP_000762903.1, encoding the Q protein in Stx2a phage from strain 00-3076, showed no significant similarity to the other Q proteins and therefore was not included. The reference corresponding to 933W phage is highlighted in blue.

The predicted sequences of Q proteins mostly clustered into two major clades (Figure 2). One group corresponded to 126-aa Q proteins and included the Q protein of the Stx2a phage of STEC strain 11128, previously described as QO111 and those from the prophages present in strains RM13516 and 155. The most extensive group comprised the Q protein of the phage 933W, named Q933, and other 144-aa Q proteins (some registered as 150-aa or 157-aa due to a larger ORF considered in the annotation). On the other hand, the predicted Q protein (273 aa) encoded by the Stx2 prophage present in STEC strain 00-3076 showed no significant similarity to the others.

Downstream *Q*, regions encoding for putative DNA methylases have been registered in some GenBank records. We identified similar sequences in all the Stx2a phages by tblastn analyses using annotated proteins against the genomic sequences of the phages. All sequences were clustered into three main groups. Two clusters corresponded to putative 50 aa-proteins and the third cluster grouped putative 352-aa proteins encoded by prophages of the strains 11128, RM13516, 155 and 00-3076.

The *stx*_2a_ sequences shared high identity (99.3 to 100%) with that present in 933W. VirulenceFinder results showed that most *stx*_2a_ sequences best matched with nucleotide sequences corresponding to the Stx2a-O157-EDL933 variant, except three that corresponded to Stx2a-O157-SF-258-98 and one to Stx2a-O113-TS17-08 (Table 1).

**Figure 3.**
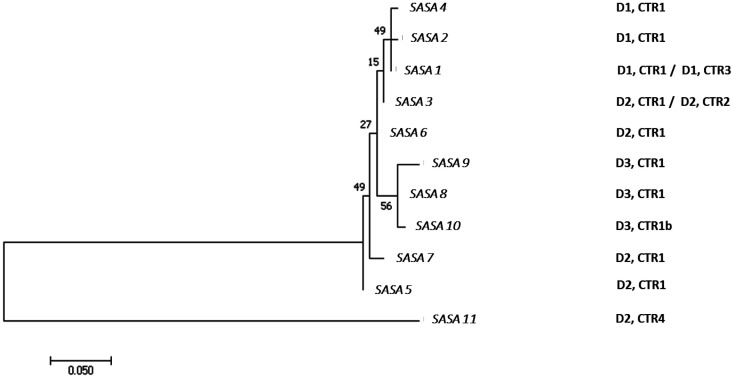
Molecular phylogenetic analysis of SASA domains identified in NanS-p proteins encoded by Stx2a phages, and their association with DUF 1737 and C-terminal domains. Protein sequences were aligned using MUSCLE. The evolutionary history was inferred by using the Maximum Likelihood method based on the Dayhoff matrix based model [64]. Initial tree(s) for the heuristic search were obtained automatically by applying Neighbor-Join and BioNJ algorithms to a matrix of pairwise distances estimated using a JTT model, and then selecting the topology with superior log likelihood value. The tree is drawn to scale, with branch lengths measured in the number of substitutions per site. The analysis involved 11 amino acid sequences of SASA domain. There were a total of 194 positions in the final dataset. Evolutionary analyses were conducted in MEGA7 [35]. Combinations of DUF 1737 and C-terminal domains present in NanS-p proteins carrying each SASA variant are listed on the right.

Comparative analyses of NanS-p identified 20 different amino acid sequences among Stx2a phages (Table 2). Sequences 1 and 16 (which only differ by the presence of an IS element in sequence 16) were the most frequent, and they were only identified in Stx2a phages carried by O157 strains. On the other hand, sequence 12 was detected in Stx2a phages present in O104 and O2 strains.

For a detailed analysis of the different NanS-p proteins, domains were identified in 933W NanS-p and used for subsequent sequence comparisons. Three sequences corresponding to the N-terminal domain (DUF1737, IPR013619/PF08410, amino acids 3-53 in 933W NanS-p) were recognized and named D1 to D3 (Table 2). D1 and D2 only differed in one amino acid and were detected in phages encoding the Stx2a-O157-EDL933 variant, while D3 was distinct and only present in the phages carried by the strains 11128, 155, RM13516 00-3076. Eleven sequences matching the catalytic esterase domain (SASA, IPR005181/PF03629, amino acids 83-276 in 933W NanS-p) were detected and named SASA1 to SASA11, this domain was the only to show homology in its whole extension across all the sequence analyzed indicating a common ancestor (Figure 3). The differences were concentrated in nine amino acid positions (Table 2). Moreover, sequence 20 (corresponding to the Stx2a prophage 86, accession NC_008464) showed lower sequence identity. The comparison of the C-terminal domain evidenced four groups of sequences (CTR1 to CTR4). Differences between CTR1 and CTR3 arose from an IS element in CTR3 that modifies the C-terminal end. All CTR1 sequences, not including CTR1b, had high similarity with the C-terminal domain of vb_24B_21 NanSp from phage Φ24B (crystallized portion, position 423-645 aa). Notably, variations were mostly concentrated in two amino acid positions (corresponding to 624 and 640 in the NanS-p sequence of vb_24B). On the other hand, the four aromatic amino acids identified in the proposed carbohydrate binding site of the C-terminal domain of the protein vb_24B_21 (Y510, Y515, Y540, F611) were highly conserved among CTR1 sequences.

**Figure 4.**
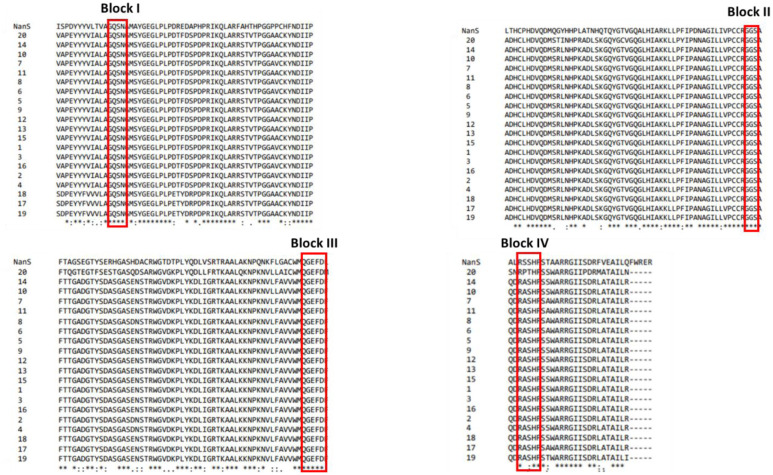
Blocks characteristic of SGNH hydrolases in bacterial NanS and in NanS-p sequences from Stx2a phages. NanS sequence from *Escherichia coli* str K-12 substr. MG1655 (NP_418729.1) and NanS-p sequences detected in this study (named 1 to 20 as listed in Table 2) were aligned using Clustal Omega [36]. Only regions that contain the blocks I to IV are shown.

NanS-p sequences shared with the bacterial NanS, from *Escherichia coli* str K-12 substr. MG1655 (NP_418729.1), the same variations in the motifs of the four blocks common to the SGNH superfamily of hydrolases (Figure 4). Only the NanS-p sequence encoded by the Stx2a prophage 86 (NanS-p sequence 20) showed two aa different in the block IV (RPTH instead of RSSH or RASH).

Between *nan*S-p and *S* genes, some annotated genomes showed other coding regions, like some encoding proteins of unknown function containing domains of the DUF826 or DUF1378 superfamilies.

Regarding predicted holins encoded by the S genes, most sequences obtained from the Stx2a phages were identical to that encoded by phage 933W or differed in one amino acid. Only one holin sequence, present in only one phage, differed 4 aa from that in phage 933W. According to the search we performed on the Transporter Classification Database (TCDB), the predicted holins for the Stx2a phages of the present study are pinholins. They showed a high identity (92.6 to 95.6%) with the bacteriophage H-19B pinholin, which belongs to the P21 holin S family (holin II superfamily).

### 3.2. Expression of nanS-p in strains belonging to different serotypes

**Figure 5.**
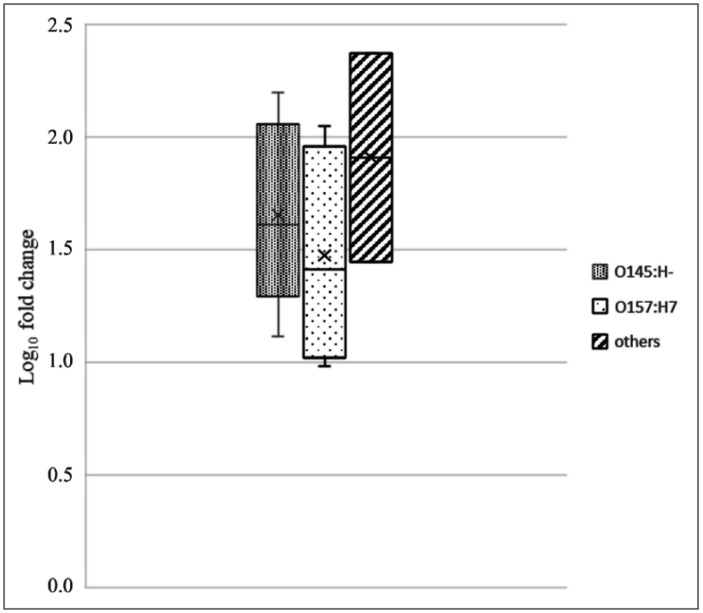
Expression of *nan*S-p in *stx*_2a_-positive STEC strains: induced expression (mitomycin C-treated cultures) relative to noninduced cultures. Box plot charts with strain results grouped by serotype. The qPCR results are expressed as log_10_ fold change values of mitomycin C-treated cultures relative to noninduced ones. Strain 355 (serotype O145:H-) did not show *nan*S-p expression and therefore was not included in the figure.

To perform a more detailed study of NanS-p, we evaluated the expression of *nan*S-p in 14 *stx*_2a_-positive STEC strains belonging to O26:H11, O91:H21, O145:H- and O157:H7 serotypes. *In silico* analysis was used to assess the PCR sequence specificity, which showed that the amplicon sequence matched only once with each of the genomes of most strains in the vicinity of the *stx*_2a_ gene. For three strains, the *stx*_2a_ gene was located on short contigs that did not include the downstream flanking sequence. The qPCR results showed that all but one strain expressed *nan*S-p, and the expression increased by 1 to 2.4 logs under mitomycin C-treatment (Figure 5).

### 3.3. NanS-p sequences in phages encoding Stx subtypes other than Stx2a

Additionally, we carried out a genome analysis of twenty-four (pro)phages harboring *stx* subtypes different from *stx*_2a_. Nineteen phages encoding Stx1a, Stx1c, Stx2b, Stx2c or Stx2d carried the *nan*S-p gene downstream the *stx* operon. Interestingly, *nan*S-p could not be identified in Stx2e, Stx2f and Stx2g phages. The NanS-p sequences showed size variation (636 to 657 aa) (Supplementary Table 2). Although the three domains were detected, sequence variability was observed: seven different N-terminal sequences, twelve SASA-domain sequences and three C-terminal sequence groups. Remarkably, the Stx1a, Stx1c and Stx2c phages had higher identity in N-terminal sequences with the Stx2a phages than the Stx2b and Stx2d phages. In addition, differences in SASA sequences were observed in similar aa positions than those identified in Stx2a phages (Supplementary Table 2). All except one of these 19 NanS-p sequences had the four blocks of the SGNH family of hydrolases described for Stx2a phages.

## 4. Discussion

Shiga toxins (Stx), the main virulence factor of STEC strains, are encoded in the genome of phages integrated into the bacterial chromosome (prophages). S.O.S induction of Stx prophages and consequent Stx expression have been demonstrated to be required for disease in animal models [41],[42]. Stx toxins and Stx phages comprise families of diverse members [6],[7]. Several studies indicate that the Shiga toxin subtype and the amount of toxin produced may be associated with severe illness, with Stx2a being a great concern for its association with severe diseases [5],[43],[44]. Conversely, less is known about other characteristics of Stx phages that could contribute to the pathogenicity of STEC strains.

Advances in sequencing technologies have provided an excellent opportunity to study Stx phages. However, the number of studies on Stx phage genomes is still limited [7]. Furthermore, despite the increased availability of genomic STEC sequences, Stx prophage sequences cannot always be fully resolved from bacterial genome sequencing if only short-read sequencing technologies have been used [45]–[47].

This study aimed to analyze the *stx* flanking region of Stx2a phages using publicly available complete sequences, including phages associated with STEC strains from different serotypes, sources and countries. As STEC strains belonging to serotype O157:H7 are most frequently isolated from humans, prophages corresponding to O157:H7 strains are found in a higher proportion in the databases. In addition, STEC genome sequences of some countries are overrepresented. Therefore, this study does not represent serotypes, sources and countries equally.

We retrieved 59 genomic sequences of Stx2a (pro)phages from databases and performed a comprehensive analysis of a region we named *Q-S*. All these genomes showed conservation of the gene order *Q*, *stx*_2a_, *nan*S-p and *S*. However, different gene alleles and allele combinations were detected. In addition, some genomes exhibited transposable insertion sequence elements (most of the IS3 family), contributing to the heterogeneity in the *Q*-*S* region.

The *Q* gene encodes an anti-terminator protein that participates in the mechanism that regulates the switch from the lysogenic to the lytic life cycle, influencing *stx* expression. Previous studies have identified *Q* gene variants, of which *Q_933_*, *Q_21_* and *Q_O111:H-_* are the best characterized [48]. However, associations between each *Q* allele and levels of Stx2 production are still not clear [37],[49]–[51]. The present study detected *stx*_2a_ genes mainly linked to genes encoding proteins Q933 and QO111:H-, and none with Q21, similar to a previous study [50]. We observed specific associations between Q and Stx2a variants: Q933 with Stx2a_O157_EDL933, QO111 with Stx2aO157-SF-258-98 and an uncharacterized Q with Stx2a-O113-TS17-08. No strong relationships were observed between serogroups and Q variants in our study.

On the other hand, the sequences of the S gene, encoding putative holins, shared high identity. Holins are part of phageś lysis system and control endolysin-mediated degradation of the bacterial peptidoglycan layer. They can be canonical holins or pinholins [52], which differ in the size and number of lesions they cause on the bacterial membrane. In contrast to canonical holins, the pinholins form much smaller and more numerous holes [53]. They are associated with SAR endolysins, which acquire an enzymatically active conformation when they are released from the membrane [52]. The predicted holins for the Stx2a phages of the present study are all pinholins, in accordance with Pinto et al. (2021), who found that most Stx phages have a pinholin among their lysis genes. In addition, our results showed a high identity between the pinholins of the Stx2a phages we studied and the pinholin of Stx1 bacteriophage H-19B, and, consequently, they belong to the P21 holin S family.

All Stx2a phage genomes harbored the *nan*S-p gene downstream *stx*_2a_, as previously observed in the reference prophage 933W from *Escherichia coli* O157:H7 strain EDL933 (where it was initially identified as ORF L0105) and in other Stx prophages from some STEC strains [54],[55]. This gene has been recognized also in non-Stx prophage genomes integrated into some *E. coli* strains, including STEC, and received its name for the homology with the chromosomally encoded O-acetylesterase NanS. Characteristically, prophage-borne genes present N- and C-terminal domains flanking the catalytic SASA domain [56]. Among the sequences of the Stx2a phages from our study, the comparison analysis of NanS-p sequences revealed some diversity in the three domains (i.e., D, SASA and CTR domains). The combination of domains named D1, SASA1 and CTR1 or CTR3 (which differs from CTR1 by the presence of an IS) was the most frequently detected among Stx2a phages carried by O157 strains. On the other hand, Stx2a phages carried by O104:H4 strains harbored the domains D2, SASA3 and CTR1. The phages that encoded QO111:H- or an untypable Q also differed in their NanS-p sequences, being the only phages with the D3 domain.

The conserved genome localization of *nan*S-p suggests that its transcription is regulated by Q protein and prompted us to evaluate its expression under basal and mitomycin C-induced conditions in a set of *stx*_2a_-positive STEC strains of our collection. The assays showed that all cultures, except one, increased *nan*S-p expression under induced conditions similar to that obtained for *stx*_2a_ in a previous study [37]. Furthermore, the exception was a strain that did not show *nan*S-p expression but had not demonstrated *stx*_2a_ expression either. Our results agree with previous studies that observed an increment in *nan*S-p expression in other inducing conditions [57]–[59], and evidence coexpression of *nan*S-p and *stx*_2a_.

Our study also showed that *nan*S-p presence was not restricted to Stx phages that carry *stx*_2a_ as it was also detected downstream of the operons encoding other Stx subtypes, supporting early findings [55]. However, *nan*S-p could not be identified in Stx2e, Stx2f and Stx2g phages. Coincidently, this gene was absent in the two Stx2f phages whose *stx*-flanking region was sequenced and analyzed by Unkmeir and Schmidt [55].

The sialate O-acetylesterase function has been demonstrated in Neu5,9Ac2 utilization for different NanS-p proteins encoded by prophages in the strains O157 EDL933 and O104 LB226692, suggesting that NanS-p could provide advantages for bacterial growth in the gut [25],[54],[60]. Analysis of NanS-p proteins in those two strains and two other O104 strains showed that NanS-p encoded by Stx1a or Stx2a phages were closely related [25],[26]. Our study provides further evidence for this, as it revealed that NanS-p sequences associated with the 59 Stx2a (pro)phages that were analyzed share close sequence similarity among them and also with those carried by phages encoding other Stx subtypes. Diversity in the catalytic domain was mainly restricted to a few amino acid positions, but higher variability was detected for the C-terminal domain. Remarkably, a study on the crystal structure of the C-terminal domain of vb_24B_21 NanS-p from phage Φ24B [61] revealed a lectin-like, jelly-roll β-sandwich fold that predictably acts as a non-catalytic carbohydrate-binding module that could assist esterase activity. Further studies are necessary to evaluate if the NanS-p subtypes differ in their activity or in binding specificity.

## 5. Conclusions

The analysis of the *Q*-*S* region in Stx2a phage genomes indicated that all sequences included the *Q*, *stx*_2a_, *nan*S-p and *S* genes. However, different alleles of these genes were observed, as well as other sequence differences. Interestingly, we found that there were groups of Stx2a phages that shared specific combinations of variants of these genes.

We identified sequence differences in each of the three domains of NanS-p esterases encoded by Stx2a phages and other Stx phages. Therefore, future studies will be necessary to evaluate if NanS-p variants differ in their activity. Our findings also suggest that phages that encode some Stx subtypes do not carry the *nan*S-p gene, but as phage genomes associated with these subtypes are underrepresented in the databases, more sequences are required to support this conclusion.
