# Supplementary material for: Characterization of the flanking region of the Shiga toxin operon in Stx2a bacteriophages reveals a diversity of the NanS-p sialate O-acetylesterase gene
Source: AIMS Microbiol. 2023 Aug 2;9(3):570–90. doi: 10.3934/microbiol.2023030 (PMC10462455; doi:10.3934/microbiol.2023030)
Supplement: Supplementary file 1 [file microbiol-09-03-030-s001.pdf]

AIMS Microbiology

AIMS Microbiology, 9(3): 570–590.
DOI: 10.3934/microbiol.2023030
Received: 05 June 2023
Revised: 18 July 2023
Accepted: 20 July 2023
Published: 02 August 2023

http://www.aimspress.com/journal/microbiology

Research article

Characterization of the flanking region of the Shiga toxin operon in Stx2a bacteriophages reveals a diversity of the NanS-p sialate O-acetylesterase gene

Stefanía B. Pascal[1,2,*], Ramiro Lorenzo[2,3], María Victoria Nieto Farías[1,2], John W.A. Rossen[4,5], Paula M. A. Lucchesi[1,2] and Alejandra Krüger[1,2]

[1] Universidad Nacional del Centro de la Provincia de Buenos Aires (UNCPBA), Facultad de Ciencias Veterinarias, CISAPA, Tandil, Buenos Aires, Argentina
[2] Centro de Investigación Veterinaria de Tandil (CIVETAN), UNCPBA-CICPBA-CONICET, Tandil, Buenos Aires, Argentina
[3] Laboratory of Neurophysiology, ULB Neuroscience Institute, Université Libre de Bruxelles (ULB), Brussels, Belgium
[4] Laboratory of Medical Microbiology and Infectious Diseases, Isala Hospital, Zwolle, The Netherlands
[5] Department of Medical Microbiology and Infection Prevention, University of Groningen, University Medical Center Groningen, Groningen, The Netherlands

* **Correspondence:** Email: spascal@vet.unicen.edu.ar.

Table S1. Strains used to evaluate *nan*S-p expression.

Serotype	Source	No. of strains	Reference
O26:H11	bovine	1	Parma et al. 2000[1]
O91:H21	bovine	1	Blanco et al. 2004[2]
O145:H-	human	4	Rivero et al. 2010[3]
O145:H-	bovine	2	Padola et al. 2004[4]
O157:H7	bovine	4	Padola et al. 2004[4]
O157:H7	hamburguer	1	Krüger et al. 2011[5]

1. Parma A, Sanz M, Blanco J, et al. (2000) Virulence genotypes and serotypes of verotoxigenic *Escherichia coli* isolated from cattle and foods in Argentina. *Eur J Epidemiol* 16: 757–762. https://doi.org/10.1023/a:1026746016896
2. Blanco M, Padola NL, Krüger A, et al. (2004) Virulence genes and intimin types of Shiga-toxin-producing *Escherichia coli* isolated from cattle and beef products in Argentina. *Int Microbiol* 7: 269–276.
3. Rivero MA, Passucci JA, Rodriguez EM, et al. (2010) Role and clinical course of Verotoxigenic *Escherichia coli* infections in childhood acute diarrhea in Argentina. *J Med Microbiol* 59: 345–352. https://doi.org/10.1099/jmm.0.015560-0
4. Padola NL, Sanz ME, Blanco JE, et al. (2004) Serotypes and virulence genes of bovine Shigatoxigenic *Escherichia coli* (STEC) isolated from a feedlot in Argentina. *Vet Microbiol* 100: 3–9. https://doi.org/10.1016/S0378-1135(03)00127-5
5. Krüger A, Lucchesi PMA, Parma AE. (2011). Verotoxins in bovine and meat verotoxin-producing *Escherichia coli* isolates: type, number of variants, and relationship to cytotoxicity. *Appl Environ Microbiol* 77: 73–79. https://doi.org/10.1128/AEM.01445-10

Table S2. Information on phages and prophages encoding Stx subtypes other than Stx2a.

Accession number	Phage name	*stx* subtype	STEC host					NanSp				
			Name	Serotype/ serogroup	Source	Isolation place	Year or period	Protein length (aa)	DUF1737 variant	SASA variant	Variable amino acids in SASA*	C-terminal region
AP012537	Stx2a_WGPS2	*stx*2c	980938	O157:H7	human	Japan	1990	645	DUF 3	SASA 15	GS**EYR**DA**A**K**LGT**	CTR 1
AP012536	Stx2a_1447	*stx*2c	1447	O157:H7	human	Japan	NA	645	DUF 3	SASA 15	GS**EYR**DA**A**K**LGT**	CTR 1
AP012530	Stx2a_F349	*stx*2c	F349	O157:H7	human	Japan	1990	645	DUF 3	SASA 12	GS**EYRE**A**AR**F**A**A	CTR 1
AP012538	Stx2a_WGPS4	*stx*2c	990281	O157:H7	human	Japan	1998	645	DUF 3	SASA 12	GS**EYRE**A**AR**F**A**A	CTR 1
AP012539	Stx2a_WGPS6	*stx*2c	990570	O157:H7	human	Japan	1998	645	DUF 4	SASA 14	**SAEYR**D**VA**KF**A**A	CTR 1
FJ188381	1717	*stx*2c	EC970520	O157:H7	NA	NA	NA	645	DUF 3	SASA 12	GS**EYRE**A**AR**F**A**A	CTR 1
CP015240	NA	*stx*2c	2011C-3911	O79:H7	human	NA	2011	654	DUF 3	SASA 16	GS**EYR**D**VA**KF**GT****	CTR 5
CP027340	NA	*stx*2b	2015C-3121	O91:H14	human	USA	2011	658	DUF 6	SASA 20	**SAEYR**DA**A**K**LA**A**	CTR 5
NZ_LOIR01000058	NA	*stx*2d	STEC 2861	O1:H20	human	Netherlands	2013	648	DUF 6	SASA 13	GS**EYRE**A**AR**FVA	CTR 1b
CP027310	NA	*stx*2d	2014C-4135	O113:H21	human	NA	2014	657	DUF 7	SASA 19	GS**EYREVA**KFVA	CTR 5
CP015020	NA	*stx*2d	28RC1	O157:H7	bovine carcass	USA	1999	648	DUF 6	SASA 17	**SAEYR**DA**AR**FVA	CTR 1
KU977419	AU5Stx1	*stx*1a	NA	O157	human	Australia	NA	646	DUF 8	SASA 14	**SAEYR**D**VA**KF**A**A	CTR 1
KU977420	AU6Stx1	*stx*1a	NA	O157	human	Australia	NA	646	DUF 8	SASA 14	**SAEYR**D**VA**KF**A**A	CTR 1
MG986485	SH2026Stx1	*stx*1a	SH2026	O157:H7	NA	NA	2015	646	DUF 8	SASA 14	**SAEYR**D**VA**KF**A**A	CTR 1
MG710528	GER2	*stx*1a	23169	O117:H7	human	United Kingdom	NA	646	DUF 2	SASA 21	**S**S**EYR**DA**A**KFVA**	CTR 1
AP005153	NA	*stx*1a	Morioka V526	O157:H7	human	Japan	NA	648	DUF 6	SASA 18	GS**EYR**D**VA**KF**A**A	CTR 1
AJ556162	BP-4795	*stx*1a	4795/97	O84:H4	NA	NA	NA	645	DUF 5	SASA 8	GS**EYR**DA**A**KF**A**A	CTR 1
AP000400	VT1-Sakai	*stx*1a	Sakai	O157:H7	human	Japan	1996	648	DUF 6	SASA 18	GS**EYR**D**VA**KF**A**A	CTR 1
CP027323	NA	*stx*1c	2013C-3033	O146:H21	human	NA	NA	636	DUF 2	SASA 22	GSDFSDA**AILA**A*	CTR 6

NA: not available in database record or associated reference.
*Positions correspond to amino acids 89, 91, 100, 102, 104, 106, 123, 124, 126, 241, 257, and 275 in 933W NanS-p sequence (GSDFSDAVKFVA). Differences with SASA1 are highlighted in bold. ** Differences were not only restricted to these positions.

AIMS Press

© 2023 the Author(s), licensee AIMS Press. This is an open access article distributed under the terms of the Creative Commons Attribution License (http://creativecommons.org/licenses/by/4.0)
